# Uptake of community-based integrated HIV and sexual and reproductive health services for young people in Zimbabwe: the CHIEDZA study

**DOI:** 10.1186/s12913-025-13635-3

**Published:** 2025-11-10

**Authors:** Victoria Simms, Ethel Dauya, Chido Dziva Chikwari, Tsitsi Bandason, Katharina Kranzer, Mandikudza Tembo, Constancia Mavodza, Aoife M. Doyle, Leyla Larsson, Owen Mugurungi, Tsitsi Apollo, Richard J. Hayes, Rashida A. Ferrand, CHIEDZA trial team

**Affiliations:** 1 MRC International Statistics and Epidemiology Group, Department of Infectious Disease Epidemiology, London School of Hygiene & Tropical Medicine, Keppel Street, London, WC1E 7HT UK; 2 The Health Research Unit Zimbabwe, Biomedical Research and Training Institute, Harare, Zimbabwe; 3 Department of Clinical Research, London School of Hygiene & Tropical Medicine, London, UK; 4 Division of Infectious Diseases and Tropical Medicine, Ludwig Maximilian University Hospital, Munich, Germany; 5 Department of Global Health and Development, Faculty of Public Health and Policy, London School of Hygiene and Tropical Medicine, London, UK; 6 AIDS and TB Unit, Ministry of Health and Child Care, Harare, Zimbabwe

**Keywords:** Youth, Young people, HIV, Sexual and reproductive health, Community-based, Zimbabwe

## Abstract

**Introduction:**

Limited engagement with health services contributes to the poorer HIV care outcomes observed in young people. We conducted a cluster-randomised trial to investigate the impact of community-based integrated HIV and sexual and reproductive health (SRH) service (CHIEDZA) for young people on HIV outcomes in three provinces in Zimbabwe. Here we examine the uptake of services.

**Methods:**

In the 12 intervention arm clusters, weekly integrated HIV and SRH services were delivered from community centres to cluster residents aged 16–24 years over 30 months. Service components included HIV testing, treatment and adherence support, management of sexually transmitted infections (STIs), menstrual health management, provision of condoms and contraception, counselling, and a tailored package of educational text messages on SRH topics. All components were optional. Fingerprint scanning was used to register clients and track their attendances and service uptake over time.

**Results:**

From April 2019-March 2022 36,991 clients attended CHIEDZA services, for a total of 78,810 visits; each centre had a median of 55 clients per day; 40.6% of clients returned for more than one visit. Overall, 75.0% of clients were female and 53.0% were aged < 20 years. Clients accessed a median 3 (IQR 2–4) service components/visit. The most popular service components for women were menstrual health products (taken up at least once by 96.5% of eligible clients), HIV testing (83.7%) and period pain management (59.9%); for men the most popular were condoms (93.9%), HIV testing (85.6%) and text messages on SRH (67.1%). The most striking difference in component uptake by age was higher uptake of condoms (43.7%) and contraception (60.3%) in women aged ≥ 20 years. In total 84.1% of eligible clients had at least one and 17.4% had > 1 HIV test. At their first visit 78.6% of eligible clients had an HIV test, and out of those who were not tested at the first visit, 28.3% later returned and were tested. HIV incidence among those with HIV status recorded at more than 1 visit was 0.72 per 100 person years (95%CI 0.53–0.98). Overall, 377 clients tested HIV positive at CHIEDZA (prevalence 1.3%) of whom 70.3% linked to care either at CHIEDZA (*n* = 234) or with other service providers (*n* = 31). An additional 1162 clients were previously diagnosed with HIV.

**Conclusions:**

An integrated HIV and SRH programme had high attendance and service uptake, with most clients accessing multiple service components per visit, including HIV testing. Provision of integrated HIV and SRH services may increase service engagement and uptake by young people and facilitate programme efficiency.

**Trial registration:**

The cluster-randomised trial was registered at www.clinicaltrials.gov (Trial registration number: NCT03719521) on 17 October 2018 (https://www.clinicaltrials.gov/study/NCT03719521).

**Supplementary Information:**

The online version contains supplementary material available at 10.1186/s12913-025-13635-3.

## Introduction

In east and southern Africa in 2021, 33% of new HIV infections occurred among young people aged 15–24 years, with an incidence three times higher in young women than young men [1]. Yet, it is estimated that only 65% of young people aged 15–24 living with HIV in the region know their HIV status, the lowest proportion of any adult age group [2].

Young people face multiple barriers to accessing health services, including judgemental attitudes of staff particularly when accessing sexual and reproductive health (SRH) services, and services that do not meet their needs [3–6]. For example, while there has been a concerted focus on HIV service provision, young people may not perceive HIV testing as a priority, being more concerned about access to other SRH services including contraception and menstrual health management [7]. Fragmented and vertical programmes with poor integration between HIV and other SRH services contribute to low demand and engagement by young people [8].

While there has been an expansion of HIV testing initiatives and scale-up of antiretroviral therapy (ART) delivery, young people in Zimbabwe continue to have disproportionately poor outcomes, including higher levels of undiagnosed HIV and lower levels of viral suppression, compared to adults [9]. HIV testing and care are offered through vertical programmes which ignore the broader needs of young people, with consequent low demand and poor engagement. This is compounded by judgemental attitudes of providers towards young people accessing HIV services [6]. Studies have reported persisting high levels of unmet need for contraception, menstrual health care, and sexually transmitted infection (STI) management among young people [10]. Combining HIV services with other SRH services might increase engagement by providing services young people actually want and lead to increased uptake of HIV testing, an entry point for HIV prevention and care. For example, integrated youth-friendly SRH for adolescent girls and young women in Malawi led to increased HIV testing uptake compared to standard of care [11]. Integrated services could also offer programmatic efficiency and a better experience for young people, and are recommended by the WHO guidelines for youth-friendly health services [12].

We conducted a trial of a multi-component service providing integrated SRH and HIV services for young people at community centres in Zimbabwe and evaluated its uptake and effectiveness in improving HIV outcomes at population level. The results of service uptake in the intervention communities are presented here and the trial outcomes will be reported separately.

## Methods

CHIEDZA (clinicaltrials.gov: NCT03719521) was a cluster-randomised trial conducted in urban (Harare, Bulawayo) and peri-urban (Mashonaland East) settings in three provinces in Zimbabwe, each province containing eight clusters randomised 4:4 to the control (existing health services) or intervention arm [13]. A cluster was a geographically defined area containing a primary care clinic and a multi-purpose community centre. One day per week, CHIEDZA staff used each community centre to deliver integrated HIV and SRH services as well as general counselling to young people aged 16–24 years residing within the cluster. Each cluster was estimated to have a population of 2000–4000 young people aged 16–24.

### CHIEDZA services

**Fig. 1:**
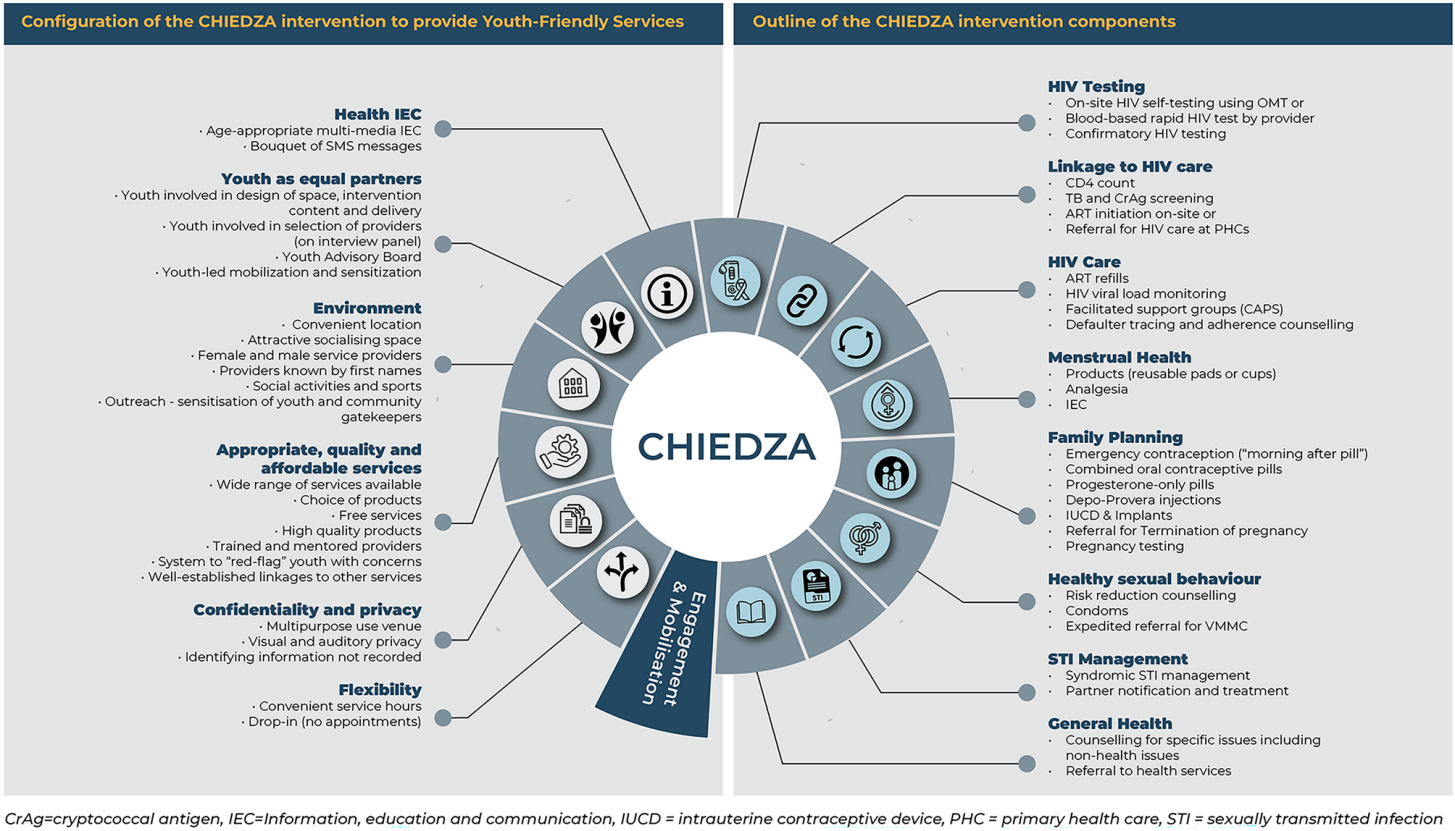
Configuration and components of the CHIEDZA service

The configuration and components of the CHIEDZA intervention are shown in Fig. 1. The intervention was co-designed with young people [10]. All service components were optional, and selected by clients from a menu provided at registration. Services were provided for 30 months per site by a multidisciplinary team of nurses, counsellors, community health workers and youth workers hired by CHIEDZA. Tasks performed only by the nurse included diagnosis and treatment of STIs, and initial consultation for a client testing HIV positive, ART initiation, and family planning prescriptions. HIV testing and other SRH services were also performed by the community health worker.

A number of studies have established that training and supervision of healthcare providers is an essential aspect of youth-friendly SRH services [14, 15]. Providers underwent a structured training programme on each service component, on principles of youth-friendliness and how to manage particularly marginalised and vulnerable groups, e.g. young people with disabilities or LGBTQI young people. Monthly briefing meetings were held with providers to discuss any complex cases, troubleshoot any operational issues, identify and address further training needs of providers and discuss any changes to service configurations that may be required.

Three methods for HIV testing were offered: rapid blood-based testing onsite by the provider, supported self-testing onsite using an oral mucosal transudate (OMT) test, or an OMT self-test to take away. If the OMT test was reactive, clients were advised to access confirmatory blood-based HIV testing either at CHIEDZA services or elsewhere. Initially, clients were eligible for an HIV test if they were not known to be HIV positive and had not had an HIV test for at least 6 months. The 6 months requirement was later dropped. Linkage to care at either a local HIV service of the client’s choice or at CHIEDZA services was offered to those who tested HIV positive or were previously diagnosed with HIV but not accessing care. Clients who were already accessing HIV care elsewhere could opt to switch to receiving their drugs and/or have an HIV viral load test at CHIEDZA services. ART was prescribed according to national guidelines and viral load was measured at six months and thereafter annually. Multimodal ART adherence support including multiple calls if clients missed visits, individualised adherence counselling and regular community adolescent peer support (CAPS) groups. Antiretroviral therapy for clients accessing HIV care at CHIEDZA was sourced from the primary care clinic in the respective cluster. Uptake of HIV care and results of the care cascade have been reported separately [16].

With respect to SRH the following were provided; at the first visit all women were eligible for menstrual health products (reusable pads or menstrual cup) unless the products were out of stock, and at later visits they were eligible for menstrual health products once every 3 months, if they wished to swap product (e.g. change from a menstrual cup to reusable pads). Products were expected to last for one year. Syndromic management of STIs including partner notification was offered, with partners treated free of charge regardless of age or whether they were resident in the cluster. A trial of STI screening was embedded within the intervention, the uptake and yield of which has been reported [17]. Information, education and counselling (IEC) materials (https://www.thruzim.org/resources) were provided combined with individualised risk reduction and general counselling. Immediate referral to a mental health intervention developed in Zimbabwe that uses a problem solving therapy approach delivered by trained lay health workers (Friendship Bench) [18] was provided for clients who had severe or complex mental health issues. Expedited referral for voluntary male medical circumcision (VMMC) was provided, but clients who accepted referral were not followed up to determine whether they completed VMMC. Youth workers also ran on-site group discussion sessions on relevant topics (e.g. condom demonstrations, how to use menstrual cups etc.). A short message message service (SMS) providing twice-weekly text messages on topics relating to SRH was introduced. This SMS intervention was adapted from one previously shown to improve knowledge and acceptability of contraception in Palestine [19].

### Data management and analysis

Each client was registered using their fingerprints, captured with SIMPRINTS software (Simprints Technology, Cambridge, UK) integrated with Android tablets (Samsung). The software converted fingerprint data into a Global Unique ID (GUID). This approach was used to enable linkage of clients’ service uptake records over time without capturing any identifying information (such as name or address) that might deter clients from accessing the service. At the first visit age, sex, birthdate and initials were recorded with service uptake, and on subsequent visits, service uptake was recorded on the tablets using SurveyCTO software (Dobility, Cambridge, MA, USA). For HIV positive clients, identifying data including name and address and contact details were collected to facilitate linkage to care and register them into the national HIV programme. Details of HIV care including ART prescription, monitoring test results, and any incident clinical events were recorded on national HIV programme patient records. HIV incidence rate was calculated using data from participants with repeat visits.

Uptake was defined as the proportion of those attendees eligible for a particular service component who took up that component. Ten service components were analysed: HIV test, condoms, menstrual health products, text messages, contraception, pain management, general counselling, VMMC referral, STI consultation (a client seeking advice about STI symptoms) and syphilis testing. Menstrual health products, analgesia and contraception were available to women only, VMMC referral to men only, and all other services to both genders.

Data were imported into an ACCESS database for cleaning and quality controlled using automated real-time quality checks. Analysis was conducted using Stata 17.0 and R 4.1.2. Sankey plots were used to represent service component uptake at the first visit related to uptake at the second visit and any subsequent visits. Chi square tests were used for descriptive statistics. Analysis at visit level was adjusted for clustering by participant. Uptake of each service component was assessed overall and by age group (16–19 vs. 20–24), sex, urban versus peri-urban setting, and whether first or repeat visit.

## Results

The intervention was operational from 1 April 2019-30 September 2021 in Harare, 1 July 2019-15 December 2021 in Bulawayo, and 14 October 2019-31 March 2022 in Mashonaland East (Supplemental Fig. 1). All components were available from the beginning of the service, except for syphilis testing (introduced on 4 December 2020) and text messages (introduced on 25 January 2021) as shown in the figure. In total 36,991 clients attended, for a total of 78,809 visits; 40.6% (*N* = 15,034) of clients returned for > 1 visit and 6.5% of clients (*N* = 2398) returned for > 5 visits. The median number of visits per client was 1 (IQR 1–2; maximum 61) and 40.6% of clients made more than 1 visit. When stratified by age group at baseline (16–19 vs. 20–24), the median and IQR of visits per person was the same (1, IQR 1–2). Each centre operated for a median of 114 days (range 108–119) over the 30-month period; once per week for 129 weeks with a two-week break at the end of each calendar year and an eight-week suspension from 27 March-17 May2020 due to national lockdown as a result of the COVID-19 pandemic. The median number of clients per community centre per day was 55 (IQR 42–70; range 1-207). Overall, 75.0% (*N* = 27,725) of clients were female and 53.0% (*N* = 19,588) were aged < 20 years at their first visit.

The median number of service components ever taken up per client was 3 (IQR 2–4, range 0–9); 3 (IQR 2–4) for females and 2 (IQR 2–3) for males, out of a maximum of 10 for females and 7 for males. The median number of services taken up in a single visit was 2 (IQR 1–3, range 0–9); 2 (IQR 1–3) for females and 1 (IQR 1–2) for males. At their first visit, the median number of services taken up was 2 (IQR 2–3) overall, 3 (IQR 2–4) for females, and 2 (IQR 1–2) for males; 84.1% of clients (74.0% of males and 87.5% of females) took up > 1 service at their first visit.

The probability of a repeat visit is shown by the number of service components taken up at the first visit (Table 1). Adjusting for sex and age category, each extra component accepted at the first visit was associated with lower odds of a repeat visit (aOR 0.94 (95%CI 0.92, 0.96), *p* < 0.001). Table 2 shows the number of clients who were ever eligible for each service component, the number and proportion who ever took it up, and the number of eligible visits at which each component was taken up.

**Table 1:** Proportion of clients who returned for a repeat visit by service component uptake at the first visit

Number of service components taken up at first visit	*N* clients who took up number of service components	*N* clients who had only 1 visit	*N* clients who returned for at least 1 repeat visit
0	385	171 (44.4%)	214 (55.6%)
1	5484	2967 (54.1%)	2517 (45.9%)
2	13,089	7723 (59.0%)	5366 (41.0%)
3	10,795	6745 (62.5%)	4050 (37.5%)
4	4808	2939 (61.1%)	1869 (38.9%)
5	1864	1077 (57.8%)	787 (42.2%)
6 or more	566	335 (59.2%)	231 (40.8%)
Total	36,991	21,957 (59.4%)	15,034 (40.6%)

**Table 2:** Eligibility and uptake of service components, at individual level and visit level

Service component	*N* clients who were ever eligible	*N* clients who ever took service	*N* eligible client visits	*N* visits when service was taken
Condoms	36,979	18,131 (49.0%)	78,768	38,124 (48.4%)
Contraception	27,634	9924 (35.9%)	55,736	19,183 (34.4%)
Counselling	36,991	1357 (3.7%)	78,809	1438 (1.8%)
HIV test	35,446	29,826 (84.1%)	71,828	38,603 (53.7%)
Menstrual health management	26,664	25,432 (95.4%)	45,698	29,200 (63.9%)
Menstrual pain management	27,725	16,475 (59.4%)	53,978	25,330 (46.9%)
SMS	15,720	9451 (60.1%)	18,951	9451 (49.9%)
STI symptoms consultation	36,991	3591 (9.7%)	78,809	4636 (5.9%)
Syphilis test	1215	1153 (94.9%)	1409	1275 (90.5%)
VMMC	5182	337 (6.5%)	10,263	363 (3.5%)

SMS: short message service. STI: sexually transmitted infection. VMMC: voluntary male medical circumcision

### Service changes

In August 2019 uptake was evaluated and some revisions were made to the package of services. Between 1 April 2019 and 28 August 2019, 1930 HIV tests were performed, of which 1731 were done by the provider on site, 171 were self-tests on site, and 26 were self-tests at home (method was not recorded for 2 tests). Of the 26 clients who self-tested at home, 14 reported their test result (13 negative, 1 indeterminate). Qualitative research identified considerable barriers to self-testing, and young people almost universally preferred provider testing [20]. As a result, the option to self-test at home was dropped at this point. At the same time the eligibility requirements for HIV testing were broadened, allowing clients to test at any time if they did not have an HIV diagnosis, to encourage wider uptake of HIV testing.

### HIV testing and treatment

At their first visit 43.6% (95%CI 43.1, 44.1) of clients reported never having had an HIV test before. Among clients aged 16–19 years 55.9% (95%CI 55.2, 56.6) had never had an HIV test before, compared to 29.8% (95%CI 29.1, 30.5) of those aged 20–24 years. The CHIEDZA service provided 38,603 HIV tests; 28,145 (72.9%) blood-based, 10,298 (26.7%) using OMT, and 160 (0.4%) indeterminate results had a sample sent to the laboratory for an ELISA test. From 29 August 2019 onwards all HIV tests were administered onsite. Out of 36,673 HIV tests in this period, 8380 (22.9%) were OMT and 28,293 (77.2%) were blood-based. Clients were more likely to request an OMT test rather than a blood-based test if it was their first visit (25.4% [95%CI 24.8–25.9], versus 17.0% [95%CI 16.3–17.7] at other visits), and if they were aged under 20, female or had never been tested before outside the CHIEDZA service (Table 3).

**Table 3:** Uptake of OMT HIV testing (versus blood-based test), STI consultation, and condoms, among eligible clients by visit type, age group, sex and whether the client had ever had an HIV test before

		Requested OMT versus BBT (from 29 August 2019 onward)		STI consultation		Took condoms	
		Proportion (95% CI)	Chi squared test result	% (95% CI)	Chi squared test result	% (95% CI)	Chi squared test result
Visit type	First	25.4% (24.8–25.9)	Χ^2^ = 302, *p* < 0.001	3.5% (3.4–3.7)	Χ^2^ = 674, *p* < 0.001	40.1% (39.6–40.6)	Χ^2^ = 1106, *p* < 0.001
	Later	17.0% (16.3–17.8)		8.0% (7.6–8.3)		55.7% (54.8–56.6)	
Age	< 20	24.8% (24.2–25.5)	Χ^2^ = 75.9, *p* < 0.001	3.6% (3.4–3.9)	Χ^2^ = 431, *p* < 0.001	42.9% (42.1–43.8)	Χ^2^ = 272, *p* < 0.001
	20+	20.9% (20.3–21.5)		7.8% (7.5–8.1)		53.0% (52.1–53.8)	
Sex	Male	21.8% (21.0-22.7)	Χ^2^ = 7.94, *p* = 0.005	4.5% (4.2–4.8)	Χ^2^ = 75, *p* < 0.001	94.3% (93.9–94.6)	Χ^2^ = 18,700, *p* < 0.001
	Female	23.3% (22.7–23.8)		6.4% (6.2–6.7)		30.1% (29.5–30.7)	
Ever had an HIV test before	No	30.8% (30.0-31.6)	Χ^2^ = 7.67, *p* = 0.006				
	Yes	29.3% (28.5–30.0)					

In total 84.1% of eligible clients (*N* = 29,826) had at least one HIV test, 17.4% had > 1 test and 1764 clients had > 2 HIV tests, to a maximum of 13 (2 male clients who tested frequently but remained negative).

**Fig. 2:**
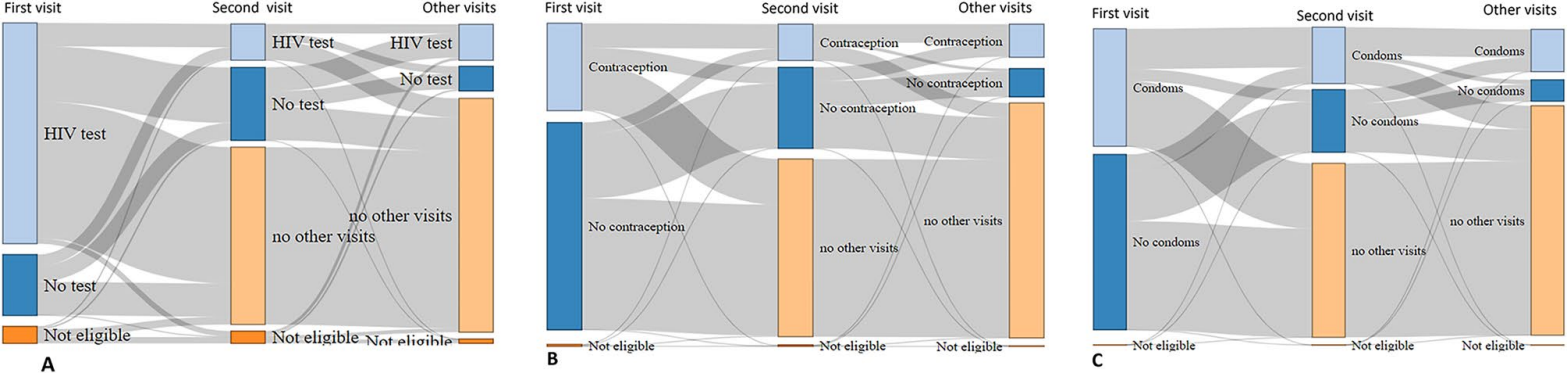
Sankey plots of (**A**) HIV test uptake; (**B**) Contraception uptake and (**C**) condom uptake, by visit

At the first visit 34,885 clients were eligible for HIV testing and 78.4% (*N* = 27338) had a test. Out of the 7547 clients who did not test at the first visit, 3436 later returned for at least one more visit, and 62.2% of them (*N* = 2136) accepted an HIV test. Meanwhile, of the 2106 clients who were not eligible for testing at their first visit, 554 (26.3%) were eligible at a later visit and 62.8% (*N* = 348) accepted a test. Testing uptake over repeat visits is shown in Fig. 2. In total, 1545 clients were never eligible for HIV testing at any visit, leaving 35,446 who were eligible. Of these, 77.1% (*N* = 27,338) had an HIV test at their first CHIEDZA visit, 7.0% (*N* = 2488) tested at a later visit and 15.9% (*N* = 5619) never tested. Uptake of HIV testing at the first visit was higher for males than females (80.6% vs. 77.6%, C^2^ = 35.8, *p* < 0.001). HIV test yield at clients’ first test was 1.3% for tests at the first visit and 1.4% for delayed tests.

Overall, 377 clients tested HIV positive at CHIEDZA (prevalence 1.3% among all those tested) and 70.3% linked to care, either at CHIEDZA (*n* = 234) or with other service providers (*n* = 31), while 1162 clients were previously diagnosed. Of these, 1090 were linked to care elsewhere and 66 transferred their care to CHIEDZA. Treatment outcomes of HIV positive clients have been reported separately [16]. HIV incidence among clients with repeat visits was 0.72 per 100 person years (95%CI 0.53–0.98).

Clients who accepted HIV testing at their first visit were more likely to receive menstrual health products, analgesia and VMMC at the same time, but less likely to receive contraception, than those who did not have an HIV test at the first visit (Table 4).

**Table 4:** Number of eligible clients who took up the service component at first visit as a percentage of those eligible, by HIV test status

Service component	HIV test at first visit	HIV test at later visit	Eligible but never tested	Never eligible/ already positive	Chi square test result
N	27,338	2484	5617	1552	
Condoms	40.2%	40.4%	37.9%	45.7%	Χ^2^ = 31.8, *p* < 0.001
Contraception	29.5%	32.6%	27.0%	35.9%	Χ^2^ = 46.6, *p* < 0.001
Counselling	1.8%	2.0%	1.9%	6.5%	Χ^2^ = 166, *p* < 0.001
Menstrual health management	94.9%	92.8%	93.5%	91.9%	Χ^2^ = 37.7, *p* < 0.001
Menstrual pain management	50.7%	43.6%	47.7%	36.7%	Χ^2^ = 129, *p* < 0.001
SMS	57.0%	57.1%	46.3%	65.0%	Χ^2^ = 33.2, *p* < 0.001
STI symptoms consultation	4.0%	1.5%	1.6%	6.1%	Χ^2^ = 136, *p* < 0.001
VMMC	4.2%	3.3%	3.2%	3.6%	Χ^2^ = 1.7, *p* = 0.63

SMS: short message service. VMMC: voluntary male medical circumcision

### STI management

There were 4636 STI consultation visits, syndromic STI treatment was given at 1679 visits and 268 of these were follow-up reviews. The consultation visits were for 3631 women and 1005 men, with syndromic treatment provided to 34.9% of women (*N* = 1268) and 40.9% of men (*N* = 411). STI consultations were more likely at repeat visits (8.0% [95%CI 7.7–8.2]) than at first visits (3.5% [95%CI 3.3–3.7]), and more common among female clients (6.4% of visits [95%CI 6.2–6.6]) than male (4.5% [95%CI 4.2–4.8]). Among women, the specific STI treatment was recorded for 1172 of the 1268 who received treatment. Among 1172 female clients managed syndromically for STIs, 59.7% were recorded as treated for vaginal discharge, 17.2% for candida, 11.4% for pelvic inflammatory disease, 7.0% for genital ulcer disease, and 3.0% for genital warts. Among the 381 men who received syndromic management for a named condition, 68.8% were treated for urethral discharge, 17.1% for genital ulcer, 8.9% for genital warts, 2.9% for epididymo-orchitis and 1.8% for a bubo.

Syphilis testing using SD Bioline was introduced as per national guidelines for STI management in December 2020, offered to all clients who had received STI syndromic management. In total 1275 syphilis tests were performed, with 62 (4.9%) positive and 3 indeterminate.

### Other service components

There were 10,263 visits by uncircumcised males, and 363 (3.5%) accepted referral for VMMC (Table 2). In 388/10,263 visits the client was living with HIV, either already diagnosed (*N* = 362), or testing positive at CHIEDZA (*N* = 26). Among clients living with HIV, 14/388 (3.6%) took up referral, and among clients not living with HIV 349/9875 (3.5%) took up referral. VMMC referral uptake was 4.0% (180/4470) at the first visit and 3.2% (183/5793) at subsequent visits.

All clients were eligible to take condoms at any visit. On 41 client visits (0.05%) condoms were out of stock. At remaining visits, clients took condoms 38,124 times (48.4% of visits). Overall uptake was 94.3% for men and 30.1% for women. Condom uptake increased with repeat visits. Out of 8428 clients who refused condoms on their first visit and later returned, 39.1% took up condoms on a subsequent visit (Fig. 2). Most clients (85.0%) who took condoms at their first visit and returned also took condoms on a later visit. Among men, uptake was 91.6% at the first visit and 96.2% at subsequent visits. Among women, condom uptake was 22.9% at the first visit and 37.1% at subsequent visits. In total 94.0% of male clients and 34.0% of female clients ever took condoms. Condom uptake was higher among women who ever took up contraception (52.0%, 5164/9923) then women who did not (23.9%, 4229/17703; χ^2^ = 2200, *p* < 0.001).

In total, 95.4% of eligible women took menstrual health products at least once. Almost all eligible clients (94.4%) took products at their first visit. At subsequent eligible visits, uptake was 25.5%. Women were eligible for period pain management (analgesia) at the first visit and then at monthly intervals. Women were eligible at 53,978 visits and took analgesics at 25,330 (46.9%). At the first visit all women were eligible for analgesia and 49.1% took it up. At later visits uptake was 44.7% (11,726/26,254 eligible visits). The maximum number of times a client took analgesia was 24, and some clients took analgesia more frequently than the monthly intervals defined by the protocol. Analgesia uptake was higher in younger women (Table 5).

**Table 5:** Proportion of eligible clients who ever took up each service component by age, sex, area type and number of visits made

Service component	Age and sex				Area type		Number of visits	
	Male < 20	Male ≥ 20	Female < 20	Female ≥ 20	Urban	Peri-urban	1	> 1
Condoms	92.0%	96.4%	25.1%	43.7%	48.9%	49.3%	37.5%	65.8%
Contraception	-	-	16.6%	57.0%	34.3%	38.8%	25.9%	50.8%
Counselling	3.1%	3.9%	3.6%	3.9%	4.4%	2.4%	1.7%	6.6%
HIV test	87.1%	83.6%	83.1%	84.2%	77.9%	95.8%	80.4%	89.5%
Menstrual health management	-	-	95.7%	95.0%	95.3%	95.5%	94.9%	96.1%
Menstrual pain management	-	-	62.6%	56.0%	66.7%	46.0%	50.7%	72.5%
SMS	58.6%	67.0%	51.3%	67.1%	58.7%	62.1%	53.2%	65.8%
STI consultation	4.8%	6.9%	13.1%	13.7%	9.1%	10.8%	3.4%	19.0%
VMMC	6.1%	7.0%	-	-	5.7%	7.9%	3.9%	9.3%

SMS: short message service. VMMC: voluntary male medical circumcision

Contraception was a repeat-use service component (Fig. 2). At their first visit 29.7% of women accepted contraception. However, 46.7% of the women who took up contraception on their first visit later returned for more visits, compared to 36.8% of those who did not take up contraception. Among those who took up contraception on their first visit and returned, the majority (77.2%) took up contraception at least once more. The median number of visits for women who ever took up contraception was 2 (IQR 1–4) compared to 1(1–2) among eligible women who did not. Among women who ever took up contraception, HIV test uptake was 86.2%, compared to 82.3% uptake for women who did not take up contraception.

From 25 January 2021, all client visits were eligible to receive text messages unless the client was already registered for it. Uptake of text message registration at eligible visits was 49.9% (9451/18,951). The most common reason clients gave for non-uptake was that they did not have a phone (4645/9500, 48.9%). Excluding these, text message service uptake at eligible visits was 66.1% (9451/14,306). In total 9451 clients (60.1% of those who attended CHIEDZA after 25 January 2021) registered for text messages.

Counselling was available at all visits and was taken up at 1438 (1.8%) visits, by 1357 unique clients. 244 red flags were identified: 112 (45.9%) for an acute mental health problem or suicidality, 56 (23.0%) for miscarriage or abortion services, 50 (20.5%) for sexual assault, 13 (5.3%) for a severe STI, (e.g. severe pelvic inflammatory disease) and 13 (5.3%) referrals to hospital for other reasons.

The most popular service components for women were menstrual health products (taken at least once by 95.4% of those eligible), HIV testing (83.7%) and analgesia (59.9%); for men the most popular were condoms (93.9%), HIV testing (85.6%) and text messages (67.1%). Among women aged ≥ 20 years, 43.7% took condoms and 60.3% took up contraception (Table 4). The group with highest uptake of HIV testing was men aged 16–19 years. All service components had higher uptake by clients who had repeat visits.

The most popular services for women living with HIV (*N* = 1375) were menstrual health products (93.3% of those eligible), text messages (65.5%), condoms (64.2%), analgesia (60.2%) and contraception (47.6%). The most popular services for men living with HIV (*N* = 164) were condoms (93.3%), text messages (66.3%), STI consultation (20.2%) and counselling (18.2%). Counselling was accepted by 17.6% of clients living with HIV versus 3.1% of those who were not HIV positive.

The peri-urban communities in Mashonaland East had higher uptake of HIV testing than the urban clusters in Harare and Bulawayo (95.8% vs. 77.9%). Menstrual pain management uptake varied widely between the provinces, from 46.0% in Mashonaland East to 57.6% in Harare and 80.8% in Bulawayo. The reasons for this diversity are unclear. Uptake of other services was broadly similar between settings.

## Discussion

Community-based integrated HIV and SRH services were in high demand among young people who attended the intervention over the study period; almost 37,000 young people received a range of integrated care, including HIV testing, STI management, contraception, menstrual health, and other service components. Each client took a median 3 components of the 10 that were analysed, and 84.1% took an HIV test. Menstrual health products and condoms were the most popular components [9].

The public health, societal and health systems benefits of integration between SRH and HIV programmes have long been recognised. Integration could improve coverage, access to and uptake of both SRH and HIV services, reduce HIV-related stigma and discrimination, and enhance programme effectiveness and efficiency as redundancies in vertical programmes are eliminated and clients’ multiple needs are addressed in one setting [21]. A systematic review of interventions linking SRH and HIV showed generally positive effects on a broad range of health outcomes and very few negative findings. SRH and HIV linkages (defined as “the bi-directional synergies in policy, programmes, services and advocacy between SRH and HIV”) were considered beneficial and feasible [22]. Integration usually concerned STI screening and treatment or family planning services, and CHIEDZA was unique in also incorporating menstrual health.

A key question is *where* and *how* to effect HIV and SRH integration to achieve engagement and utilisation; there is a paucity of evidence-based approaches to inform service delivery. Young people are infrequent users of health facilities, and out-of-facility approaches may be an important avenue to reach them [23]. There has been widespread emphasis on youth centres, but little evidence for their effectiveness, with youth centre visitors being usually a relatively small proportion (10–20%) of the young people in the community, and most visitors taking up mainly social activities with low uptake of SRH services [24].

Recently, there has been a renewed call to catalyse action towards integration of HIV and SRH services to curb the HIV epidemic, highlighting the importance of meaningfully engaging with young people to design appropriate services [25]. A strength of CHIEDZA was that it centred young people, involving them in the design, configuration and delivery of the intervention as well as in community engagement [10]. Young people selected community centres as the service delivery site because they were easy to access and multipurpose (used for a variety of community activities).

Most clients took up more than one component, demonstrating the benefits of integration. High-demand SRH services such as condoms and menstrual health products could potentially serve as a “hook” for engaging young people and provide an opportunity to offer HIV testing. For example, there was very high demand for menstrual health care with 95% of women taking up a product and 50% accessing analgesia, probably attributable to substantial unmet need in the community [7]. The intervention incorporated both HIV care (including ART initiation, treatment monitoring and adherence support) and HIV prevention (including condoms, risk reduction counselling and referral for VMMC) so that following HIV testing, clients could be channelled to further appropriate services. The intervention also catered for those who were already diagnosed and accessing HIV care elsewhere, who could choose to either have their ART pick-up through CHIEDZA and/or access the range of SRH services available, addressing a recognised gap in HIV programmes that have often focused exclusively on ART delivery [26]. Such a service also provided a platform for STI screening which was introduced and has been reported separately [17]. This integration of HIV and STIs was recommended in the WHO Global Health Sector Strategy 2022–2030 [27].

A key facet of the model was incorporating choice in the delivery and type of commodities, to promote autonomy and person-centredness. For example, studies that have used community-based HIV testing for young people have found alternative testing sites to be acceptable and feasible, with uptake among young people who have never previously tested [3]. Within CHIEDZA, a choice of menstrual health products and contraceptive methods were offered; for HIV testing both provider-delivered and self-testing were available, but the latter was dropped due to very low uptake. The Yathu Yathu study in Zambia, which also evaluated community-based SRH services for young people, reported similar findings with clients preferring provider testing using a finger-prick sample over oral self-testing [28]. An ongoing evaluation embedded within CHIEDZA showed that there was little awareness of the concept of self-testing, and young people were concerned about how they would link to care and also how they would deal with a positive test result without provider support [20]. Similarly, in qualitative interviews CHIEDZA staff reported desire for provider support as a barrier to digital-supported HIV self-testing, along with lack of self-agency and challenges with digital literacy, privacy or the technology [29]. In contrast, previous studies where self-testing kits were distributed in communities showed high uptake among young people and were a particularly appropriate method for hard-to-reach groups such as young men [30, 31]. This difference underscores the importance of eliciting and responding to the context-specific preferences of young people.

Clients could access as few or as many services as they wanted at each visit, providing flexibility. Clients who took up more service components at their first visit were slightly less likely to return for a repeat visit, which may indicate that their needs were fully met at the first visit. However, some services, such as the oral contraceptive pill or Depo-Provera injections, required regular repeat visits. Uptake of services including HIV testing was higher for clients who made multiple visits. Clients who refused an HIV test at their first visit often accepted one at a later visit. Repeat visits and greater familiarity may have encouraged clients’ confidence and reduced fears associated with HIV testing [28].

High uptake of HIV testing was achieved through community-based integrated HIV-SRH services. Similar uptake was observed in the Yathu Yathu study which evaluated the effectiveness of SRH service hubs for young people with incentivisation to use services ( [32]: in the 5-month pilot implementation phase, 76.2% of clients had an HIV test at the hub [28]. While HIV testing uptake within CHIEDZA services was high, yield was low and the majority (1162/1539, 75.6%) of young people living with HIV who encountered CHIEDZA already knew their status. It is possible that young people at highest risk of undiagnosed HIV either did not attend CHIEDZA or may not have accepted HIV testing. The fact that HIV testing was offered to all attendees regardless of their level of risk may have contributed to the low yield. The higher uptake of HIV testing in parei-urban Mashonaland East may be partly attributable to the fact that Mashonaland East was the last province to deliver CHIEDZA, and benefitted from the learning from the other two provinces in how to optimise HIV testing.

Alongside integrated services and choice, training and ongoing supervision of providers was a core component to ensure that high-quality services were delivered. Training focused specifically on how to provide care for LGBQTI clients and clients with disabilities, on maintaining confidentiality, non-judgemental interactions, and ensuring person-centred and “youth-friendly” care. Clients highly valued these service attributes alongside the integration of services [33]. There is evidence that interventions to make services more youth-friendly often result in increased uptake of services [34]. The most frequently assessed indicators for “youth-friendliness” are accessibility, staff competency, and confidentiality and privacy, but these indicators reflect basic standards of care and are not specific to young people’s needs [35].

Counselling was available for all clients, but the uptake of counselling was relatively low, although other studies of the young urban population in Zimbabwe have found high prevalence of symptoms of anxiety and depression [36]. Clients may not have perceived that they would benefit from counselling. Education on mental health literacy, recognition of symptoms and mental health first aid may be beneficial [37].

Three quarters of clients were female, and some of the most popular service components (menstrual health products, analgesia and contraception) were only accessible to women. Active attempts were made to engage men and provide services that would appeal to them but the proportion of male attendees remained unchanged. Condoms and text messages were the only services that were more popular among men than women. Males aged 16–19 had the highest uptake of HIV testing although they are the group with lowest HIV prevalence of all those eligible for CHIEDZA services [9]. Non-health-related service components may be more appealing to young men and more likely to meet a felt need.

The study had several strengths. Service operation took place across a large number of communities, for an extended period (30 months). Fingerprint identification enabled us to monitor clients over repeated visits and track individual-level service uptake over time, without requiring clients to provide personal details about themselves. The intervention was flexible enough to incorporate pragmatic changes based on an embedded ongoing evaluation and logistic constraints, while maintaining the ethos of youth-centred SRH services.

A limitation is that very limited socio-demographic data were collected from clients, such as their relationship status or previous history of care. This was a deliberate decision to maintain confidentiality and establish trust with clients. The study took place in urban and peri-urban settings and cannot be generalised to rural areas. There was no referral for pre-exposure prophylaxis (PrEP) or post-exposure prophylaxis (PEP), and no assessment for risk of HIV acquisition was done. Addition of these services would more comprehensively address the needs of young people.

The COVID-19 pandemic and lockdowns in 2020 and 2021 had profound impacts on CHIEDZA services, which have been reported [38], as have the implications of the pandemic for contraception [39] and menstrual health [7] service delivery. Prior to May 2020 the CHIEDZA programme included a range of activities such as football, music and dancing, and social spaces, in keeping with a youth-friendly approach. These activities were closed to comply with infection prevention guidelines, leaving only the health provision services [38]. In addition, opening hours were shortened due to government restrictions, and the number of clients allowed on site at any time was limited. As a result some young people were turned away from the service and others may have been deterred from attending.

## Conclusions

An integrated HIV and SRH programme had high attendance and uptake, with the average client accessing two service components per visit, and 84% of clients accepting an HIV test. Integrated services could potentially increase engagement, but careful attention is needed in design and configuration of services and, crucially, the training and skills of health care providers.

## Supplementary Information

Below is the link to the electronic supplementary material.

Supplementary Material 1: Supplemental Fig. 1: Service components offered and number of clients by province over time

## Data Availability

The data is available from the study team at request, with further use contingent on approval from the Medical Research Council of Zimbabwe.
