# Supplementary figures and images for: Uptake of community-based integrated HIV and sexual and reproductive health services for young people in Zimbabwe: the CHIEDZA study
Source: BMC Health Serv Res. 2025 Nov 10;25:1459. doi: 10.1186/s12913-025-13635-3 (PMC12604306; doi:10.1186/s12913-025-13635-3)

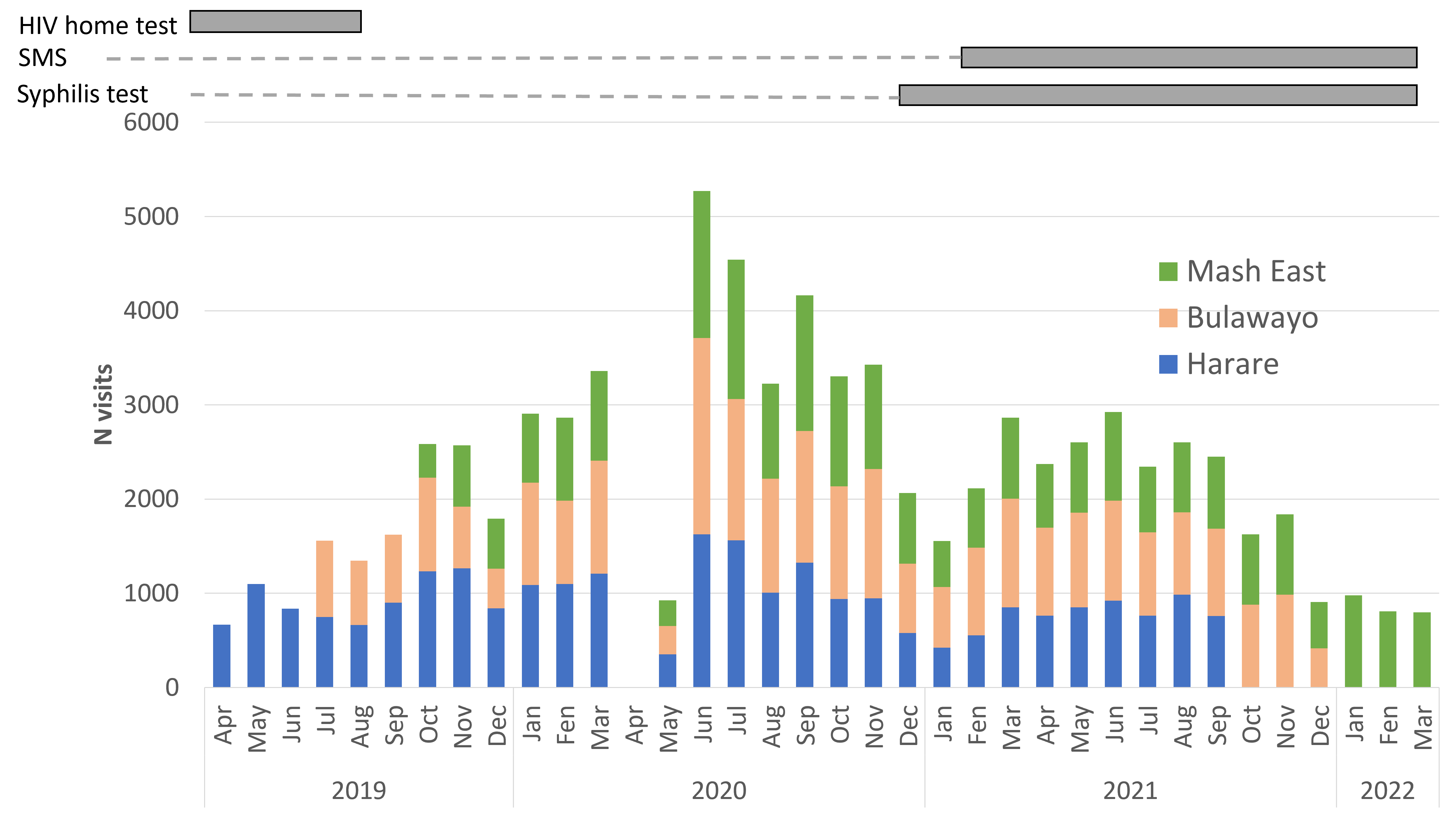

Supplement: Supplementary file 1 — Supplementary Material 1: Supplemental Fig. 1: Service components offered and number of clients by province over time [file 12913_2025_13635_MOESM1_ESM.tif]
